# Inhibition of IL-1β by Aliskiren Improved Renal AQP2 Expression and Urinary Concentration Defect in Ureteral Obstruction and Release

**DOI:** 10.3389/fphys.2019.01157

**Published:** 2019-09-13

**Authors:** Shan Hu, Haixia Xie, Renfei Luo, Pinning Feng, Qiaojuan Liu, Mengke Han, Yonglun Kong, Xuenong Zou, Weidong Wang, Chunling Li

**Affiliations:** ^1^Institute of Hypertension, Zhongshan School of Medicine, Sun Yat-sen University, Guangzhou, China; ^2^Department of Clinical Laboratory, The First Affiliated Hospital, Sun Yat-sen University, Guangzhou, China; ^3^Guangdong Provincial Key Laboratory of Orthopedics and Traumatology, The First Affiliated Hospital, Sun Yat-sen University, Guangzhou, China; ^4^Department of Nephrology, The Seventh Affiliated Hospital, Sun Yat-sen University, Shenzhen, China

**Keywords:** renin inhibition, urinary concentration, ureteral obstruction, IL-1β, AQP, inflammasome, renin

## Abstract

We previously demonstrated that ureteral obstruction is associated with a urinary concentrating defect and reduced expression of renal aquaporins (AQPs), in which the renin–angiotensin system (RAS) may play an important role. The aims of the present study were to examine whether the renin inhibitor aliskiren could prevent the reduction in AQP expression and improve the urinary concentrating capacity in mice with bilateral ureteral obstruction (BUO) and BUO release. BUO was performed for 24 h, and BUO release was performed for 1 (B-R1D) or 3 days (B-R3D) with or without aliskiren treatment. Aliskiren prevented polyuria and decreased urine osmolality induced by B-R3D. In mice with BUO and BUO release, aliskiren attenuated the reduction in AQP2 protein and mRNA expression in the obstructed kidneys. B-R3D increased the protein expression of NLRP3 inflammasome components ASC, caspase-1, and interleukin-1β in the obstructed kidneys, which was markedly prevented by aliskiren. Moreover, the NF-κB inhibitor Bay 11-7082 blocked NLRP3 inflammasome activation and attenuated the decrease in AQP2 protein expression in primary cultured rat inner medullary collecting duct cells treated with angiotensin II. These results indicate that the renin inhibitor aliskiren increases water channel AQP2 expression at least partially by suppressing NLRP3 inflammasome activation in the obstructed kidneys of mice with BUO and BUO release.

## Introduction

Obstruction nephropathy is associated with reduced glomerular and tubular function as well as long-term impaired urinary concentrating capacity. Release of both unilateral and bilateral ureteral obstruction (UUO and BUO) causes marked changes in the glomerular-tubular balance and so-called postobstructive diuresis (Frokiaer and Zeidel, 2011). Clinical observations have indicated that patients with obstructive nephropathy experience massive vasopressin-insensitive polyuria after the release of the obstruction, suggesting that BUO is associated with nephrogenic diabetes insipidus. We previously demonstrated that ureteral obstruction (both bilateral and unilateral) and its release in rats is associated with significant downregulation of aquaporin (AQP) protein expression in the kidneys, providing a molecular basis for the urinary concentrating defect (Li et al., 2001, 2003). The expression of the vasopressin-regulated collecting duct water channel AQP2 was reduced in BUO for 24 h in rats, which persisted 24–48 h after the release of the obstruction, accompanied by marked postobstructive polyuria (Frokiaer et al., 1996b). Rats with UUO showed downregulated AQP2 expression similar to that observed in rats with BUO (Li et al., 2003). This finding indicates that local/intrarenal factors may play important roles in the downregulation of AQP expression in obstructive nephropathy.

Recently, the intrarenal renin–angiotensin system (RAS) in the kidneys has been demonstrated to play an important role in cellular homeostasis. For example, local RAS activation in the kidneys may induce structural and functional changes in tubular epithelial cells independent of those elicited by the classical renin–angiotensin endocrine system (De Mello and Frohlich, 2014). Angiotensin II (Ang II) is synthesized *de novo* in the kidneys and may directly modulate renal hemodynamic and tubular transport (Braam et al., 1993; Reams et al., 1993). Obstructed kidneys have been reported to show increased intrarenal Ang II levels (Okabe et al., 2015), which may play a role in the reduction of the glomerular filtration rate (GFR) and renal blood flow (RBF) in ureteral obstruction. Reportedly, angiotensin-converting enzyme inhibitors (ACEIs) or Ang II type 1 receptor blockers (ARBs) partly prevent the reduction in GFR and RBF (Frokiaer et al., 1996a) and improve the expression and intracellular trafficking of renal AQP2 and several key sodium transporters in ureteral obstruction (Jensen et al., 2006, 2009; Topcu et al., 2007), indicating that Ang II contributes to the urinary concentrating defect of obstructed kidneys. In addition, Ang II infusion has been reported to induce the infiltration of monocytes and the release of inflammatory factors in the kidneys (Crowley et al., 2010), probably due to activation of the intracellular NF-κB pathway (Muller et al., 2000). RAS blockade attenuates the expression of an array of cytokines and growth factors, which potentially impair renal function in rats with UUO (Ishidoya et al., 1995; Wu et al., 2010; Soliman et al., 2011; Wang W. et al., 2015). Compared with ACEIs or ARBs, direct renin inhibitors (DRIs), such as aliskiren, can block the RAS at an early stage in the cascade and potently suppress the formation of Ang I and Ang II via both ACE and non-ACE pathways (Segall et al., 2007). Aliskiren is therefore an alternative option to inhibit RAS activation beside ACEi and ARB. A recent clinical trial showed that aliskiren improved renal and systemic hemodynamics (Kwakernaak et al., 2017), providing an effective treatment in hypertensive, cardiovascular, and renal diseases.

NOD-, LRR-, and pyrin domain-containing 3 (NLRP3) inflammasome is a cytosolic signaling macromolecular complex comprising a sensor molecule [NOD-like receptors (NLRs)], the adaptor apoptosis-associated speck-like protein containing a CARD (ASC), and the effector protease caspase 1, which processes pro-interleukin (IL-1β) into its mature form IL-1β that causes inflammation and tissue damage (Mangan et al., 2018). Recent studies have suggested that NLRP3 inflammasome and downstream cytokines contribute to several types of kidney disease, including crystalline nephropathy (Mulay et al., 2013; Wang et al., 2018), obstructive nephropathy (Wang W. et al., 2015), and obesity-related kidney diseases (Ke et al., 2018). We previously demonstrated that IL-1β directly inhibits AQP2 expression in the collecting duct principal cells of the kidneys (Wang W. et al., 2015), suggesting that inflammatory cytokines downregulate the expression of water channels and sodium transporters, resulting in altered water and sodium regulation in the kidneys. The present study aimed to investigate whether aliskiren alleviates the abnormal water regulation in the kidneys of mice with BUO and BUO release and attenuates the reduction in water channel expression and the activation of NLRP3 inflammasome.

## Materials and Methods

### Reagents

For semiquantitative immunoblotting and immunocyto chemistry, previously characterized affinity-purified polyclonal antibodies to AQP2 and AQP3 were used (Wang et al., 2018). Antibodies to AQP1 were obtained from BOSTER (Wuhan, China); antibodies to IL-1β, p-NF-κB p65, and NF-κB p65 from Cell Signaling Technology; caspase-1 from Abcam; antibodies to ASC from Santa Cruz; and NLRP3 from Novus. Human IL-1β was obtained from Peprotech and Bay 11-7082 was purchased from Sigma–Aldrich. Ang II and valsartan were purchased from MCE (Shanghai, China).

### Animals and Treatments

All animal procedures were approved by the Animal Care and Use Committee of Sun Yat-sen University. In brief, 8-week-old male C57BL/6 mice were purchased from the animal facility center of Sun Yat-sen University (Guangzhou, China), were maintained on a light/dark (12/12 h) cycle at 24°C, and received food and water *ad libitum* before experimentation.

To prepare for the surgery to establish BUO, the mice were anesthetized with pentobarbital sodium and placed on a heated table to maintain their rectal temperature at 37–38°C. A midline abdominal incision was then made to expose both ureters, and the ureters were occluded by ligating a 5-0 silk suture at the midportion. For BUO release, ureters were occluded by vascular clamps (#14120, World Precision Instruments Inc., Sarasota, FL, United States) at the midportion. Mice were intraperitoneally injected with aliskiren twice per day (40 mg/kg body weight/day). BUO was released by removing the vascular clamps after 16 h. By using this technique, the ureters of the mice could be completely occluded for 16 h without any subsequent impairment of ureteral function. The mice were sacrificed 1 or 3 days after BUO release.

Mice were allocated to the protocols indicated below. Age- and time-matched sham-operated controls were prepared and observed in parallel with each BUO group and BUO release (B-R) group. *Protocol 1* included BUO for 24 h (BUO), BUO with aliskiren injection (BUO + Ali), and sham-operated (Sham) groups (*n* = 9 in each group). *Protocol 2* included BUO for 16 h followed by 1-day release (B-R1D), and B-R1D with aliskiren injection (B-R1D + Ali) and Sham groups (*n* = 9 in each group). *Protocol 3* included BUO for 16 h followed by 3-day release (B-R3D), and B-R3D with aliskiren injection (B-R3D + Ali), and Sham groups (*n* = 9 in each group).

All mice were maintained in metabolic cages during experimentation. Their daily water and food intake was monitored. Mice were placed in metabolic cages immediately after BUO or BUO release. Urine samples for clearance studies were collected (BUO-1D and BUO-3D) over 24-h periods. On the sacrifice day, all mice were anesthetized with pentobarbital sodium, and both kidneys were removed from each animal and prepared for protein, mRNA measurement, or histologic analysis.

### Blood and Urine Chemistry

Urine was collected and clearance studies were performed during periods throughout the study. At the end of each protocol, blood samples were collected into heparinized tubes for the determination of serum creatinine and osmolality when the mice were sacrificed. The osmolality of urine and serum was determined by freezing-point depression (OM 806, Osmometer, Loser, Germany). The serum and urine creatinine were determined by using an ELISA kit according to the manufacturer’s instructions (BioAssay System, United States). Creatinine clearance Ccr (μL/min/g BW) is calculated based on the following formula: Ccr = [urinary creatinine concentration (mmol/L) × urine output (μL/min/g)]/[plasma creatinine concentration (mmol/L)]. Urine and serum potassium concentrations were analyzed by using a flame photometer.

### Electrophoresis and Immunoblotting

At the sacrifice day, mice were anesthetized with pentobarbital sodium, and kidneys were frozen in liquid nitrogen immediately after removal. Tissue was minced finely and homogenized in dissecting buffer (0.3 M sucrose, 25 mM imidazole, 1 mM EDTA, pH 7.2, containing the following protease inhibitors: 8.5 μM leupeptin, 1 mM phenylmethylsulfonyl fluoride). This homogenate was centrifuged at 4,000 × *g* for 15 min at 4°C. The supernatants were assayed for protein concentration using the BCA method (Pieces, Rockford, IL, United States). Gel samples were made from this pellet. Samples of membrane fractions were run on 12% polyacrylamide minigels. After transferring by electroelution to PVDF membranes, blots were blocked with 5% milk in PBS-T (80 mM Na_2_HPO_4_, 20 mM NaH_2_PO_4_, 100 mM NaCl, 0.1% Tween 20, pH 7.5) for 1 h and incubated with primary antibodies overnight at 4°C. After being washed with PBS-T, the blots were incubated with horseradish peroxidase-conjugated secondary antibody (Pieces, Rockford, IL, United States). After a final washing as above, corresponding secondary antibodies were visualized using enhanced chemiluminescence (Pierce, Rockford, IL, United States). The signals were quantified with a chemiluminescence detector and the accompanying densitometry software (UVP, Upland, CA, United States).

### Immunohistochemistry and Immunofluorescence

The kidneys from mice were fixed by retrograde perfusion via the left ventricle with 0.01 M PBS buffer. The left kidney blocks containing all kidney zones were dehydrated and embedded in paraffin. For immunoperoxidase microscopy, sections (4 μm thick) cut from paraffin-embedded kidney samples were used for immunostaining of AQP2 (Wang et al., 2018). Briefly, after dewaxing and rehydration, a microwave pretreatment in citrate buffer (pH 6.2) was performed to unmask antigens present in the renal tissue. Tissue sections were then incubated overnight at 4°C with primary antibodies. After rinsing in PBS, slides were exposed to the secondary antibody for 1 h. The sections were later washed with PBS, followed by incubation with diaminobenzidine for 10 min. The microscopic examination was carried out by using a Leica DM2000 light microscope (Leica, Heidelberg, Germany). For sections prepared for immunofluorescence, AQP2 (Santa Cruz, sc-515770) and ASC (Abcam, ab64808) were used for primary antibodies. Secondary fluorescent antibodies were obtained from Santa Cruz. Fluorescence microscopy was carried out on a Leica DMI4000B fluorescence inverse microscope (Heidelberg, Germany).

### RNA Extraction and Quantitative Real-Time PCR

Total RNA was extracted from the kidneys according to the manufacturer’s instructions for Trizol reagent (Invitrogen, CA, United States). Total RNA (500 ng) was used for reverse transcription using PrimeScript^®^ RT reagent Kit Perfect Real Time kit (Takara Bio Inc., Japan). The cDNA was used for quantitative real-time PCR (qRT-PCR) analysis using SYBR^®^ Premix Ex Taq^TM^ (Perfect Real Time, Takara Bio Inc., Japan). Target mRNA was determined using the comparative cycle threshold method of relative quantitation. The calibrator sample was selected from PBS-treated tissue, and GAPDH was used as an internal control. Primer sequences used are provided in Table 1.

**TABLE 1:** Primer sequences for RT-PCR (mouse).

**Target gene**	**Primer sequence**	**Target gene**	**Primer sequence**
GAPDH F	TGACCTCAACTACATGGTCTACA	AGT F	ATGCACAGATCGGAGATGACT
GAPDH R	CTTCCCATTCTCGGCCTTG	AGT R	CATGCAGGGTCTTCTCATTCAC
AQP2 F	GGACCTGGCTGTCAATGCTC	Renin F	CACACTCAGCAGTACGGACTACGT
AQP2 R	GCGGGCTGGATTCATGGAG	Renin R	CAGTGGGTGGTGGGATGTC
IL-1β F	GAAATGCCACCTTTTGACAGTG	ReninR F	CCGTAAACGCCTGTTTCAAG
IL-1β R	TGGATGCTCTCATCAGGACAG	ReninR R	TAGCACTTGCAGTTCGGAGA
KIM-1 F	ACATATCGTGGAATCACAACGAC	NGAL F	ACGGACTACAACCAGTTCGC
KIM-1 R	ACTGCTCTTCTGATAGGTGACA	NGAL R	AATGCATTGGTCGGTGGGG
V2R F	GCTGTGGCTCTGTTTCAAGTG		
V2R R	CCAGGATCATGTAGGAAGAGGC		

*GAPDH, glyceraldehyde-3-phosphate dehydrogenase; AQP, aquaporin; IL, interleukin; AGT, angiotensinogen; reninR, renin receptor; KIM-1, kidney injury molecule-1; NGAL, neutrophil gelatinase-associated lipocalin; V2R, vasopressin type 2 receptor; F, forward; R, reverse.*

### Primary Rat Inner Medullary Collecting Duct Cell Culture Studies

Primary cultures of renal inner medullary collecting duct (IMCD) cells were generated with a modification of a previous study (Luo et al., 2019). In brief, Wistar rats (8–10 weeks) were killed by cervical dislocation, and kidneys were quickly removed under sterile condition. The renal medulla was dissected, minced, and digested for 60 min in 10 mL of medium (DMEM) containing 0.2% collagenase type I, 0.2% hyaluronidase, and 0.025% trypsin-EDTA at 37°C with shaking. The IMCD cells were cultured in 6-well plates for 48 h. In protocol 1, the IMCD cells were treated with or without IL-1β (5 ng/mL) for 6 h. In protocol 2, the IMCD cells were pretreated with or without Bay 11-7082 (10^–6^ M) or valsartan (10^–6^ M) for 30 min, and then incubated with Ang II (10^–9^ M) for 24 h. After incubation, cells were then centrifuged at 1,000 rpm for 5 min, the supernatant was discarded, and the pellet was resuspended in the modified medium (DMEM, and 100 U/mL penicillin G-streptomycin sulfate).

### Measurement of Ang II

Urinary and plasma Ang II concentration was determined using an Ang II magnetic particle-based chemiluminescence assay kit (Autobio Diagnostics Co., Ltd.).

### Statistical Analysis

Results are presented as the means ± SE. Data were analyzed by ANOVA and Student–Newman–Keuls test for multiple comparisons. Statistical significance was accepted at the *P* < 0.05 level. Values represent means ± SE of three independent sets of experiments in primary cell culture studies.

## Results

### Aliskiren Prevented the Downregulation of AQP2 Expression in the Obstructed Kidneys of BUO Mice

**FIGURE 1:**
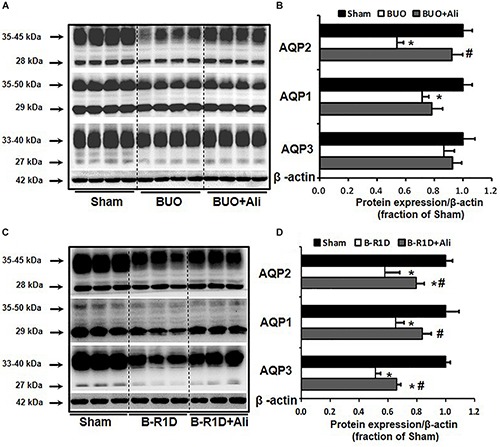
Aliskiren treatment prevented the reduction in water channel AQP2 expression in the obstructed kidneys of BUO and B-R1D mice. Semi-quantitative immunoblots probed with anti-AQP2, -AQP1, -AQP3, and -β-actin antibodies in the whole kidneys of BUO mice **(A)** and in the whole kidneys of B-R1D mice **(C)**. Corresponding densitometric analyses of AQP2, AQP1, and AQP3 protein expression corrected for β-actin in BUO mice **(B)** and in B-R1D mice **(D)**. Sham, sham-operated mice; BUO, bilateral ureteral obstruction for 24 h; BUO + Ali, BUO mice treated with aliskiren; B-R1D, bilateral ureteral obstruction for 16 h followed by 1-day release; B-R1D + Ali, B-R1D mice treated with aliskiren. ^∗^*p* < 0.05 compared with the sham group; #*p* < 0.05 compared with the BUO/B-R1D group.

Bilateral ureteral obstruction was performed for 24 h in mice treated with or without aliskiren. As shown in Figure 1, western blot analyses revealed that the abundance of renal AQP2 and AQP1 proteins was significantly lower in the obstructed kidneys of BUO mice than in the kidneys of Sham mice, and the reduction in AQP2 protein expression was markedly prevented by aliskiren treatment (Figures 1A,B). Notably, AQP3 protein expression was not significantly different between the three groups (BUO, BUO + Ali, and Sham).

### Aliskiren Prevented Polyuria and Reduction in AQP2 Expression in the Obstructed Kidneys of B-R1D Mice

Release of BUO causes postobstructive diuresis (Frokiaer et al., 1996b). As shown in Table 2, urine output was dramatically increased and urine osmolality was markedly decreased in mice with BUO that was released for 1 day (B-R1D), both of which were found to be prevented by aliskiren treatment in B-R1D + Ali mice (Table 2). The abundance of renal AQP1, AQP2, and AQP3 proteins was significantly lower in the obstructed kidneys of B-R1D mice than in the kidneys of Sham mice, and the reduction was prevented by aliskiren treatment in B-R1D + Ali mice (Figures 1C,D).

**TABLE 2:** KIM-1 and NGAL mRNA levels (arbitrary units) in BUO, B-R1D, and B-R3D kidney with or without aliskiren treatment.

	**KIM-1**	**NGAL**
Sham	1 ± 0.3	1 ± 0.3
BUO	446 ± 45.2^∗^	167 ± 51.5^∗^
BUO + Ali	236 ± 3.2^∗^^#^	152 ± 10.6^∗^

Sham	1 ± 0.1	1 ± 0.2
B-R1D	152 ± 6.9^∗^	112 ± 21.4^∗^
B-R1D + Ali	125 ± 13.4^∗^	34.5 ± 5.8^∗^^#^

Sham	1 ± 0.2	1 ± 0.2
B-R3D	19.4 ± 2.6^∗^	16.6 ± 3.9^∗^
B-R3D + Ali	18.9 ± 1.6^∗^	15.3 ± 1.4^∗^

*KIM-1, kidney injury molecule-1; NGAL, neutrophil gelatinase-associated lipocalin; Sham, sham-operated groups; BUO, bilateral ureteral obstruction for 24 h; B-R1D, bilateral ureteral obstruction for 16 h followed by 1 day release; B-R3D, bilateral ureteral obstruction for 16 h followed by 3 days release. ^∗^*p* < 0.05 compared with Sham group; ^#^*p* < 0.05 compared with obstructed kidney group.*

### Aliskiren Prevented the Reduction in AQP2 Expression in the Obstructed Kidneys of B-R3D Mice

**FIGURE 2:**
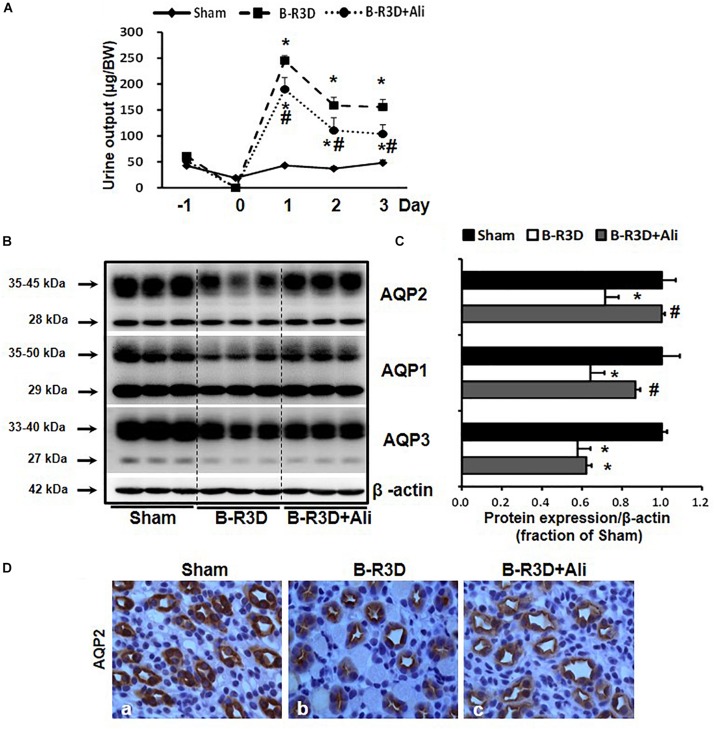
Aliskiren treatment prevented polyuria and the reduction in water channel AQP2 and AQP1 expression in the obstructed kidneys of B-R3D mice. **(A)** Urine output was collected from sham, B-R3D, and aliskiren-treated mice. **(B)** Semi-quantitative immunoblots probed with anti-AQP2, -AQP1, -AQP3, and β-actin antibodies. **(C)** Corresponding densitometric analyses of the AQP2, AQP1, and AQP3 protein expression corrected for β-actin. **(D)** Immunohistochemistry of AQP2 in the kidneys of aliskiren-treated or non-treated B-R3D mice. Sham, sham-operated groups; B-R3D, bilateral ureteral obstruction for 16 h followed by 3 days release; B-R3D + Ali, B-R3D group with aliskiren treatment. ^∗^*p* < 0.05 compared with sham group; ^#^*p* < 0.05 compared with B-R3D groups.

Urine output persistently increased in mice with BUO that was released for 3 days (B-R3D) compared with that in Sham mice (Figure 2A). This increase was prevented by aliskiren treatment in B-R3D + Ali mice (Figure 2A). The abundance of AQP2 protein was significantly lower in the obstructed kidneys of B-R3D mice than in the kidneys of Sham mice (Figures 2B,C). This decrease was significantly reversed by aliskiren treatment in B-R3D + Ali mice (Figures 2B,C). Immunohistochemistry revealed that the overall decrease in AQP2 labeling intensity observed in the inner medulla of the kidneys of B-R3D mice was clearly prevented by aliskiren treatment in B-R3D + Ali mice (Figure 2D). The expression of both AQP1 and AQP3 proteins was also markedly downregulated in B-R3D mice, but aliskiren treatment prevented the reduction in only AQP1 protein expression in B-R3D + Ali mice (Figures 2B,C).

### Aliskiren Inhibited IL-1β Production in the Obstructed Kidneys of B-R3D Mice

**FIGURE 3:**
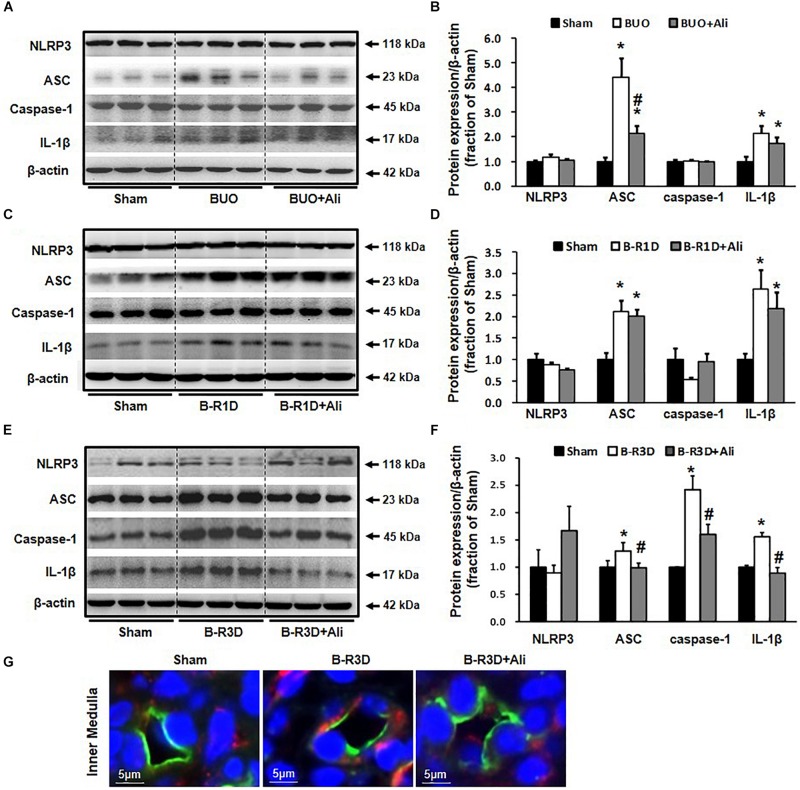
Expression of inflammasome components in the obstructed kidneys of BUO, B-R1D, and B-R3D mice. **(A,C,E)** Semi-quantitative immunoblots probed with anti-NLRP3, -ASC, -caspase-1, and -IL-1β antibodies. **(B,D,F)** Corresponding densitometric analyses of the NLRP3, ACS, caspase-1, and IL-1β protein expression corrected for β-actin. **(G)** Immunofluorescence staining of AQP2 (in green) and ASC (in red) in principal cells of the collecting duct in the kidneys of B-R3D and B-R3D + Ali mice. NLRP3, NOD-like receptor pyrin domain containing 3; ASC, apoptosis-associated speck-like protein containing a caspase recruitment domain; Caspase-1, an evolutionarily conserved enzyme that proteolytically cleaves other proteins; IL-1β, interleukin-1β; Sham, sham-operated groups; BUO, bilateral ureteral obstruction for 24 h; BUO + Ali, BUO group with aliskiren treatment; B-R1D, bilateral ureteral obstruction for 16 h followed by 1 day release; B-R1D + Ali, B-R1D group with aliskiren treatment; B-R3D, bilateral ureteral obstruction for 16 h followed by 3 days release; B-R3D + Ali, B-R3D group with aliskiren treatment. ^∗^*p* < 0.05 compared with Sham group; ^#^*p* < 0.05 compared with OBS groups.

Inflammasome activates caspase 1, which proteolytically activates the pro-inflammatory cytokines IL-1β and IL-18 (Martinon et al., 2002). Production of cleaved IL-1β was therefore examined in this study. The protein expression of the inflammasome components ASC and cleaved IL-1β (17 kDa) was increased in the obstructed kidneys of BUO and B-R1D mice; in comparison, BUO + Ali and B-R1D + Ali mice showed mild, insignificant decreases in ASC and IL-1β expression because of aliskiren treatment (Figures 3A–D). The expression of ASC, caspase-1, and IL-1β proteins was markedly upregulated by approximately 1.3-, 2.5-, and 1.8-folds, respectively, in the kidneys of B-R3D mice, whereas aliskiren treatment markedly blocked this upregulation in B-R3D + Ali mice (Figures 3E,F). Immunofluorescence showed ASC-positive staining in principal cells of the collecting ducts. The increased staining of ASC specks in principal cells of B-R3D + Ali mouse kidneys was abolished by aliskiren treatment (Figure 3G).

**FIGURE 4:**
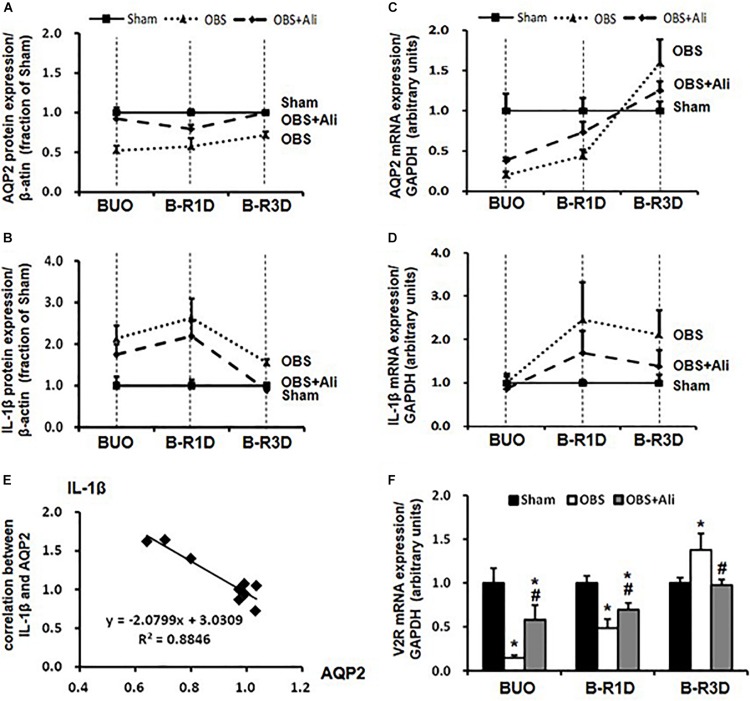
Time-course changes in AQP2 **(A,C)** and IL-1β **(B,D)** protein and mRNA levels in the kidneys of BUO, B-R1D, and B-R3D mice. **(E)** Correlation between AQP2 and IL-1β protein expression in the obstructed kidneys of B-R3D mice (*n* = 9). **(F)** V2R mRNA levels in the kidneys of BUO, B-R1D, and B-R3D mice. IL-1β, interleukin-1β; V2R, vasopressin type 2 receptor; Sham, sham-operated groups; BUO, bilateral ureteral obstruction for 24 h; BUO + Ali, BUO group with aliskiren treatment; B-R1D, BUO for 16 h followed by 1 day release; B-R1D + Ali, B-R1D group with aliskiren treatment; B-R3D, BUO for 16 h followed by 3 days release; B-R3D + Ali, B-R3D group with aliskiren treatment; OBS, obstructed group; OBS + Ali, obstructed group with aliskiren treatment.

The time-course changes in cleaved IL-1β and AQP2 protein and mRNA expression during BUO, as well as BUO followed by release for 1–3 days (B-R1D and B-R3D), are shown in Figure 4. The renal abundance of AQP2 protein was maintained at the lowest level in the obstructed kidneys of the BUO group compared with the abundance in the two other groups. Notably, in the BUO-R1D + Ali and B-R3D + Ali groups, AQP2 protein expression was nearly maintained as that in the Sham group (Figure 4A), indicating that aliskiren treatment attenuated the reduction in AQP2 protein expression in the obstructed kidneys. In addition, aliskiren inhibited IL-1β expression by approximately 20–50% of that in the BUO, B-R1D, and B-R3D groups (Figure 4B), suggesting that local inflammatory cytokines (e.g., IL-1β) associated with kidney obstruction contribute to the downregulation of water channel AQP2 expression. AQP2 mRNA expression gradually increased in BUO, B-R1D, and B-R3D mice. At day 3 after release, AQP2 mRNA expression in the obstructed kidneys was higher than that in the kidneys of Sham mice, likely indicating the occurrence of a compensatory response after release (Figure 4C). Consistent with the expression pattern of IL-1β protein, IL-1β mRNA expression was higher in the obstructed kidneys than in the kidneys of Sham mice (Figure 4D). Figure 4E shows a potential linear correlation between IL-1β and AQP2 protein expression in the Sham, B-R3D, and B-R3D + Ali groups.

Interleukin-1β reduced binding of vasopressin to its receptor *in vitro* (Grinevich et al., 2004), indicating impairment of vasopressin action in the kidney during inflammation. Vasopressin-V2R and its corresponding signaling pathway are important for regulation of AQP2; we therefore examined mRNA expression of V2R in the obstructed kidneys. Compared with sham mice, mRNA levels of V2R dramatically decreased to about 15% in BUO and 50% in BUO-1D mice, which was prevented partially by aliskiren treatment (Figure 4F). In the obstructed kidneys of BUO-3D mice, V2R mRNA was increased by 37% compared to sham mice (Figure 4F). The pattern of V2R gene changes in the obstructed kidneys was similar to that seen in changes of AQP2 expression. Next we examined whether IL-1β directly affects V2R expression in primary cultured IMCD cells (protocol 1). IL-1β markedly decreased the mRNA level of V2R by 25% (0.75 ± 0.05 vs. 1.00 ± 0.05 in controls, *p* < 0.05, *n* = 6 in controls and *n* = 7 in IL-1β treatment group). These data suggested that inflammatory factors (e.g., IL-1β) induced by ureteral obstruction not only downregulate AQP2 expression, but also decrease V2R gene expression, potentially affecting the V2R intracellular signaling pathway.

### RAS in the Obstructed Kidneys of BUO, B-R1D, and B-R3D Mice

**FIGURE 5:**
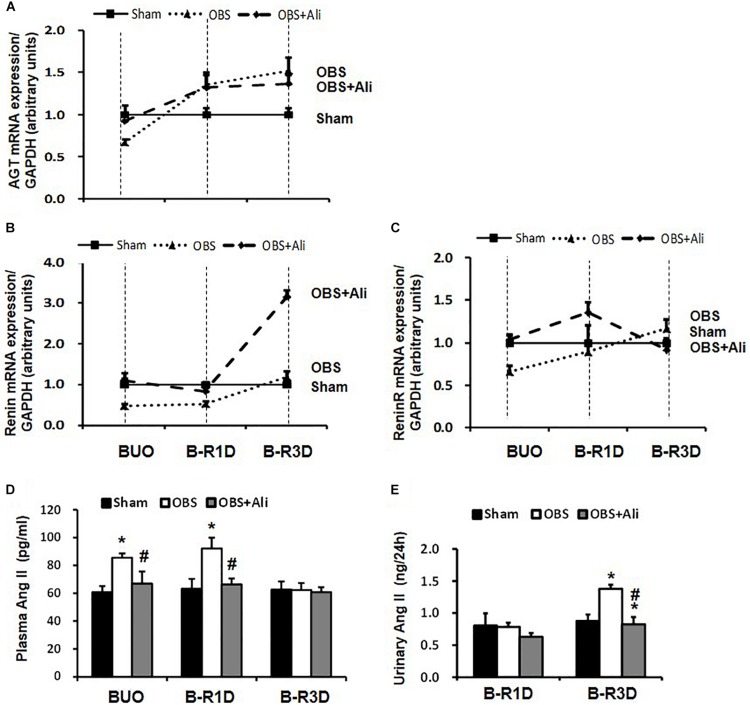
Dynamic changes in AGT, renin, and renin receptor mRNA expression in the kidneys and plasma and urinary Ang II levels in aliskiren-treated or non-treated BUO, B-R1D, and B-R3D mice. **(A,B,C)** Analysis of AGT, renin, and renin receptor mRNA expression by real-time PCR. **(D,E)** Plasma and urinary Ang II levels in aliskiren-treated or non-treated BUO, B-R1D, and B-R3D mice. Ang II, angiotensin II; AGT, angiotensinogen; ReninR, renin receptor; Sham, sham-operated groups; BUO, bilateral ureteral obstruction for 24 h; BUO + Ali, BUO group with aliskiren treatment; B-R1D, BUO for 16 h followed by 1 day release; B-R1D + Ali, B-R1D group with aliskiren treatment; B-R3D, BUO for 16 h followed by 3 days release; B-R3D + Ali, B-R3D group with aliskiren treatment; OBS, obstructed group; OBS + Ali, obstructed group with aliskiren treatment. ^∗^*p* < 0.05 compared with Sham group; ^#^*p* < 0.05 compared with B-R3D groups.

Gene expression levels of several RAS components in the obstructed kidneys were examined in BUO, B-R1D, and B-R3D mice. Angiotensinogen (AGT) mRNA expression reduced by 30% in BUO mice and increased by 1.4- and 1.5-folds in B-R1D and B-R3D mice, respectively, compared with that in the sham mice (Figure 5A). Renin mRNA levels were also reduced in BUO mice but returned to sham levels in B-R3D mice. Renin mRNA levels dramatically increased after aliskiren treatment, probably due to the inhibition of renin activity by aliskiren (Figure 5B). Renin receptor mRNA levels decreased in BUO mice and gradually returned to sham levels after BUO release in B-R1D and B-R3D mice (Figure 5C). Notably, aliskiren treatment had negligible effects on renin receptor mRNA expression in the obstructed kidneys (Figure 5D). Plasma and urinary levels of Ang II were measured. As shown in Figure 5D, plasma Ang II levels were ∼42% higher in BUO mice and ∼46% higher in B-R1D mice than those in sham mice – these increases were attenuated by aliskiren treatment. After release by 3 days, the plasma Ang II concentration returned to baseline level (Figure 5D). In B-R1D mice, urinary Ang II levels remained unchanged, whereas aliskiren treatment slightly reduced urinary Ang II concentration, although it did not reach statistical significance. Urinary Ang II levels were ∼60% higher in B-R3D mice than those in sham mice, which were completely suppressed by aliskiren treatment in B-R3D + Ali mice (Figure 5E).

Interestingly the observed changes of RAS components were associated with tubular injuries. mRNA levels of kidney injury molecule-1 (KIM-1) and neutrophil gelatinase-associated lipocalin (NGAL), two markers of tubular injuries, were dramatically increased in the obstructed kidneys. Aliskiren treatment decreased KIM-1 and NGAL mRNA levels, respectively (Table 2), indicating intervention by RAS blockade may ameliorate tubular injuries associated with obstruction. In B-R3D mice both KIM-1 and NGAL mRNA levels decreased markedly when compared to BUO or B-R1D, suggesting a potential recovery of tubular function, coincidently paralleling with recovered protein expression of AQPs. Aliskiren treatment is sometimes associated with hyperkalemia (Parving et al., 2012). In BUO and BUO + Ali mice, plasma potassium concentration showed an approximately 1.6-fold increase, but it markedly reduced after release of ureteral obstruction. Three days after release, plasma potassium concentrations returned to sham levels, but about a 20% increase was still found in B-R3D mice with aliskiren treatment (Table 3), likely due to inhibition of Ang II production.

**TABLE 3:** Renal functional data in BUO, B-R1D, and B-R3D mice with or without aliskiren treatment.

	**Sham**	**BUO**	**BUO + Ali**	**Sham**	**B-R1D**	**B-R1D + Ali**	**Sham**	**B-R3D**	**B-R3D + Ali**
BW (g)	22.5 ± 1.0	22.5 ± 0.6	22.8 ± 0.5	22.0 ± 0.8	22.7 ± 0.6	22.4 ± 0.5	21.2 ± 0.4	21.6 ± 0.3	20.5 ± 0.5
*P*_K_ (mmol/L)	3.3 ± 0.09	5.8 ± 0.33^∗^	5.5 ± 0.15^∗^	3.5 ± 0.05	4.5 ± 0.48^∗^	4.6 ± 0.34^∗^	3.5 ± 0.04	3.7 ± 0.02	4.3 ± 0.04^∗^^#^
*C*_Cr_ (μL/min/g bw)				4.93 ± 1.03	2.27 ± 0.51^∗^	2.70 ± 0.20^∗^	4.91 ± 0.13	4.32 ± 0.54	3.67 ± 0.20
UO (μl/24 h/g bw)				34.0 ± 4.7	241.1 ± 9.7^∗^	206.4 ± 10.9^∗^^#^	48.2 ± 5.2	155.7 ± 10.2^∗^	103.7 ± 14.1^∗^^#^
*U*_Osm_ (mOsm/kg⋅H_2_O)				2403 ± 244	413 ± 19^∗^	531 ± 26^∗^^#^	2684 ± 321	1215 ± 103^∗^	1333 ± 135^∗^

*BW, body weight; *P*_*K*_, plasma potassium; *C*_*Cr*_, creatinine clearance rate; UO, urine output; *U*_*Osm*_, urine osmolality; Sham, sham-operated groups; BUO, bilateral ureteral obstruction for 24 h; B-R1D, bilateral ureteral obstruction for 16 h followed by 1 day release; B-R3D, bilateral ureteral obstruction for 16 h followed by 3 days release. ^∗^*p* < 0.05 compared with Sham group; ^#^*p* < 0.05 compared with obstructed kidney group.*

### Bay 11-7082 and Valsartan Prevented the Reduction in AQP2 Expression in Ang II-Treated Primary Cultured Rat Inner Medullary Collecting Duct Cells

**FIGURE 6:**
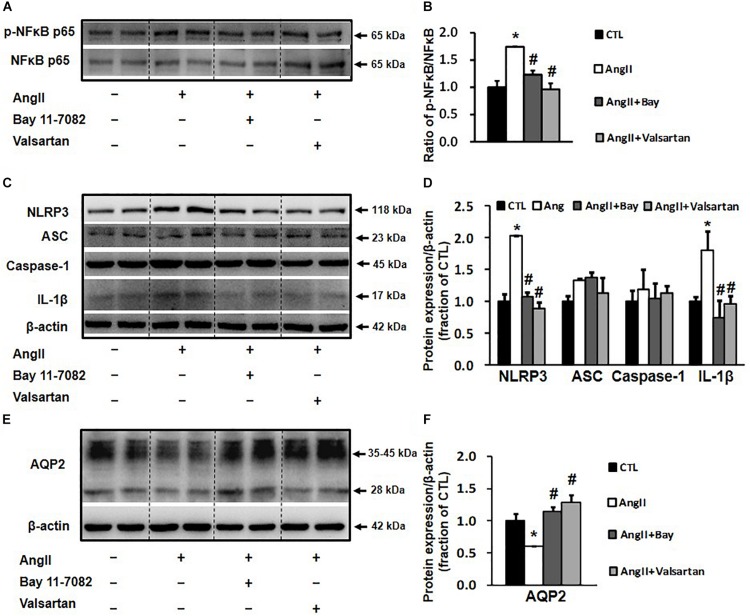
Western blot analysis of NLRP3 inflammasome components and AQP2 in primary cultured rat IMCD cells incubated with Ang II (1 nM) and co-treated with the NF-κB inhibitor Bay 11-7082 (100 μM) or the AT1 receptor blocker valsartan (100 μM). **(A)** Semi-quantitative immunoblots probed with anti-p-NF-κB and NF-κB antibodies. **(B)** The p-NF-κB/NF-κB ratio. **(C)** Semi-quantitative immunoblots probed with anti -NLRP3, -ACS, -caspase-1, -IL-1β and -β-actin antibodies. **(D)** Corresponding densitometric analyses of NLRP3, ACS, caspase-1 and IL-1β protein expression corrected for β-actin. **(E)** Semi-quantitative immunoblots probed with anti-AQP2 and -β-actin antibodies. **(F)** Corresponding densitometric analyses of AQP2 protein expression corrected for β-actin. NF-κB, transcription factors of the nuclear factor κB; p-NF-κB, phosphorylated-NF-κB (Ser536); NLRP3, Nod-like receptor pyrin domain containing 3; ASC, apoptosis-associated speck-like protein containing a caspase recruitment domain; Caspase-1, an evolutionarily conserved enzyme that proteolytically cleaves other proteins; IL-1β, interleukin-1β; ^∗^*p* < 0.05 compared with control group; ^#^*p* < 0.05 compared with Ang II group.

The activation of NLRP3 inflammasome has been associated with the NF-κB signaling pathway (Kong et al., 2016a); we therefore investigated whether the inhibition of NLRP3-associated inflammatory response by blocking the NF-κB pathway affects AQP2 protein expression. In primary cultured rat IMCD cells, Ang II treatment increased the p-NF-κB p65/NF-κB p65 ratio by 1.8-folds, which was markedly reduced by the NF-κB inhibitor Bay 11-7082 (Figures 6A,B). Ang II treatment also increased the protein expression of the NLRP3 inflammasome components NLRP3 and IL-1β and markedly decreased that of AQP2, both of which were prevented by Bay 11-7082 (Figures 6C–F). This finding suggests that Bay 11-7082 improves AQP2 expression via its local anti-inflammatory role. In order to examine a role of local Ang II in induction of inflammation that reduces AQP2 protein expression, ARBs valsartan, but not aliskiren that inhibits Ang II production, was therefore used to block effects of Ang II in primary cultured IMCD cells. As expected, valsartan showed effects that inhibited IL-1β and increased AQP2 expression, similar to those of Bay 11-7082 on Ang II-treated IMCD cells (Figure 6).

## Discussion

In this study, we demonstrated that aliskiren, a DRI, prevented the reduction in AQP2 mRNA and protein expression in the obstructed kidneys of BUO, B-R1D, and B-R3D mice. Ureteral obstruction caused RAS activation in the kidneys and NLRP3 inflammasome-induced IL-1β production, which is likely to have contributed to the downregulation of AQP2 expression in mice with BUO and BUO release.

Long-term impairment of urinary concentrating ability was observed in mice with BUO and BUO release (Frokiaer and Zeidel, 2011), which is probably attributable to reduced AQP2 protein expression in the obstructed kidneys (Frokiaer et al., 1996b). Recent evidence has demonstrated that in addition to systemic RAS, local RAS is involved in renal water metabolism and blood volume regulation (Wang F. et al., 2015). ACEIs, ARBs, and DRIs have been shown to prevent renal fibrosis and inflammation induced by UUO, suggesting that RAS is activated in ureteral obstruction (Kaneto et al., 1994; Ishidoya et al., 1995; Kellner et al., 2006; Wu et al., 2010; Sakuraya et al., 2014). RAS activation also plays a role in the downregulation of AQP2 protein expression associated with ureteral obstruction. In several studies, ARB significantly prevented the decrease in AQP2 protein expression in the postobstructed kidneys (Jensen et al., 2006; Topcu et al., 2007), prevented the downregulation of vasopressin V2 receptor complex, and reversed the obstruction-induced inhibition of stimulated cAMP generation in the inner medulla of obstructed kidneys (Jensen et al., 2009). The DRI aliskiren blocks RAS at an early stage in the RAS cascade and has been widely used for treating cardiovascular diseases. We previously demonstrated that RAS suppression with aliskiren prevented the downregulation of AQP2 expression in the UUO kidneys; consistent with the results of previous studies (Eskild-Jensen et al., 2007) demonstrating that RAS blockade by ARB prevented the reduction in AQP2 expression after partial UUO in pigs.

Increased intrarenal Ang II secretion actively contributes to afferent vasoconstriction and reduction in the GFR following the release of ureteral obstruction. AGT mRNA expression was increased in the kidneys after BUO release, suggesting an activation of intrarenal RAS in these conditions, which was supported by increased urinary Ang II excretion at the third day after release. In the kidney of B-R3D + Ali mice, renin mRNA levels were dramatically increased after aliskiren treatment, probably due to a compensatory response, as observed in other animal studies (Wu et al., 2010).

Interestingly, local RAS activation in the kidneys was associated with downregulated AQP2 protein expression and increased urine output after BUO release, which was also prevented by aliskiren, as aliskiren decreases Ang II production by suppressing renin activity. The mechanism by which intrarenal RAS activation decreases AQP2 protein expression is not completely clear. One suggested mechanism is that Ang II stimulates inflammatory responses associated with obstruction. Ang II may activate several transcriptional factors (e.g., NF-κB) modulating gene expressions of proinflammatory factors within the nucleus of tubular cells. This ultimately leads to tubulointerstitial inflammation, fibrosis, and permanent loss of renal function, which may continue to progress after the obstruction has been relieved (Frokiaer and Zeidel, 2011).

Upon activation, NF-κB is translocated into the nucleus, where its p65 subunit is phosphorylated; this induces the transcription and translation of proinflammatory cytokines, e.g., IL-1β (Tak and Firestein, 2001). A recent study demonstrated marked increases of the transcript abundances of NF-κB family genes in the kidney of rats with lithium-induced NDI, which is believed to contribute to downregulation of AQP2. Induction of NF-κB signaling and an inflammatory-like response may repress AQP2 transcription in lithium-induced NDI (Sung et al., 2019), since NF-κB binds to the AQP2 gene promoter and represses AQP2 transcription induced by vasopressin or hypertonicity (Hasler et al., 2008).

Our data showed that Ang II not only stimulated the NF-κB inflammatory pathway in rat IMCD cells, as indicated by increased NF-κB expression and phosphorylation, but also activated NLRP3 inflammasome and promoted IL-1β protein expression in rat IMCD suspensions. NF-κB may be involved in the regulation of NLRP3 inflammasome transcription (Qiao et al., 2012) and NLRP3 inflammasome participates in NF-κB-mediated inflammatory processes (Kong et al., 2016b). Interestingly, both Bay 11-7082 and valsartan inhibited Ang II-induced NF-κB activation and NLRP3/IL-1β protein expression (Figure 6), indicating potential links between the intrarenal RAS, NLRP3 inflammasome, and NF-κB pathways during inflammatory responses. Importantly, NLRP3 or NF-κB suppression by Bay 11-7082 or valsartan at least partially prevented the reduction in AQP2 expression after Ang II treatment in IMCD cells, which is likely attributable to the inhibition of IL-1β production. Changes in gene and protein expression during BUO and BUO release clearly showed a causal relationship between IL-1β and AQP2 expression (Figure 4). In general, at individual time points after BUO and BUO release, markedly increased IL-1β expression was associated with reduced AQP2 protein and gene expression. These alterations in IL-1β and AQP2 expression were prevented by aliskiren, indicating that RAS blockade increases AQP2 expression by suppressing inflammation. It is noted that there may be several other mechanisms by which IL-1β regulates AQP2. IL-1β downregulated V2R gene expression and reduced binding of vasopressin to V2R (Grinevich et al., 2004), thus inhibiting the V2R intracellular signaling pathway and downregulating AQP2 gene and protein expression.

Protein expressions of AQP1 and AQP3 were also downregulated in BUO and BUO-R, consistent with earlier studies (Li et al., 2001), which was partially prevented by aliskiren if at all. The molecular mechanism of AQP3 and AQP1 regulation in the kidney is not fully understood. Altered AQP2 expression is not always paralleled with AQP3 in some conditions. The ARB candesartan prevented the reduced AQP2 protein expression in obstructed kidneys of rats with bilateral ureter obstruction and release, but the reduction of AQP3 expression persisted (Jensen et al., 2006), indicating a different signaling pathway in AQP3 regulation from AQP2 in the kidney. Interestingly, aliskiren showed a slightly better protection in AQP1 expression than AQP3 in BUO and BUO-R, but the underlying mechanism was not investigated. Recovery of AQP1 in the kidney may in fact protect tubules from further injuries induced by BUO and BUO-R. In endotoxin-related acute kidney injury, mice with AQP1 knockout showed severe tubular injury histologically and functionally compared with wild-type mice, indicating a protective role of AQP1 in tubular injury and healing (Wang et al., 2008).

Although aliskiren increased AQP2 protein expression in BUO and BUO release, likely by inhibiting the expression of inflammatory cytokines – in particular, IL-1β production induced by intrarenal RAS activation – aliskiren may also directly stimulate AQP2 expression and thus water metabolism in the kidneys. Our recent study demonstrated that aliskiren upregulates AQP2 protein expression in IMCD principal cells via cAMP-PKA pathways. Aliskiren can also improve lithium-induced urinary concentration defect and polyuria (Lin et al., 2017). Thus, there are multiple mechanisms in the kidneys contributing to the observed protective role of aliskiren in UUO, BUO, and BUO release.

In summary, the results of this study show that RAS blockade with the renin inhibitor aliskiren prevented drastic reduction in AQP2 protein expression, probably by inhibiting inflammatory responses, especially IL-1β expression, in the kidneys of mice with BUO and BUO release. The results indicate that intrarenal RAS activation plays a potential role in inflammasome maturation during ureteral obstruction. In addition to blocking RAS activation, inhibition of both NLRP3 inflammasome and NF-κB pathways may be a promising therapeutic strategy for obstructive nephropathy.

## Data Availability

All datasets generated for this study are included in the manuscript and/or the supplementary files.

## Ethics Statement

The animal study was reviewed and approved by the Committee on the Management and Use of Laboratory Animals, Sun Yat-sen University.

## Author Contributions

SH, HX, RL, PF, QL, MH, and YK performed the experiments. SH, RL, WW, and CL analyzed the data. SH, HX, WW, and CL interpreted the results of experiments. SH and CL prepared the figures. SH, WW, and CL drafted the manuscript. SH, XZ, WW, and CL edited and revised the manuscript. WW and CL approved the final version of the manuscript.

## Conflict of Interest Statement

The authors declare that the research was conducted in the absence of any commercial or financial relationships that could be construed as a potential conflict of interest.
